# Process evaluation in the field: global learnings from seven implementation research hypertension projects in low-and middle-income countries

**DOI:** 10.1186/s12889-019-7261-8

**Published:** 2019-07-16

**Authors:** Felix Limbani, Jane Goudge, Rohina Joshi, Marion A. Maar, J. Jaime Miranda, Brian Oldenburg, Gary Parker, Maria Amalia Pesantes, Michaela A. Riddell, Abdul Salam, Kathy Trieu, Amanda G. Thrift, Josefien Van Olmen, Rajesh Vedanthan, Ruth Webster, Karen Yeates, Jacqui Webster, The Global Alliance for Chronic Diseases, Process Evaluation Working Group, Alfonso Fernandez Pozas, Anushka Patel, Arti Pillay, Briana Cotrez, Carlos Aguilar Salinas, Caryl Nowson, Claire Johnson, Clicerio Gonzalez Villalpando, Cristina Garcia-Ulloa, Debra Litzelman, Devarsetty Praveen, Diane Hua, Dimitrios Kakoulis, Ed Fottrell, Elsa Cornejo Vucovich, Francisco Gonzalez Salazar, Hadi Musa, Harriet Chemusto, Hassan Haghparast-Bidgoli, Jean Claude Mutabazi, Jimaima Schultz, Joanne Odenkirchen, Jose Zavala-Loayza, Joyce Gyamfi, Kirsty Bobrow, Leticia Neira, Louise Maple-Brown, Maria Lazo, Meena Daivadanam, Nilmini Wijemanne, Paloma Almeda-Valdes, Paul Camacho-Lopez, Peter Delobelle, Puhong Zhang, Raelle Saulson, Rama Guggilla, Renae Kirkham, Ricardo Angeles, Sailesh Mohan, Sheldon Tobe, Sujeet Jha, Sun Lei, Vilma Irazola, Yuan Ma, Yulia Shenderovich

**Affiliations:** 1 Centre for Health Policy, School of Public Health, Faculty of Health Sciences, University of the Witwatersrand, 27 St Andrews Rd Parktown, Private Bag X3 Wits, Johannesburg, 2050 South Africa; 2 The George Institute for Global Health, Sydney, New South Wales Australia; 3 Sydney Medical School, University of Sydney, Sydney, New South Wales Australia; 4 Faculty of Medicine, University of New South Wales, Sydney, New South Wales Australia; 5 Faculty of Medicine, Northern Ontario School of Medicine, Laurentian University, Sudbury, ON Canada; 6 CRONICAS Center of Excellence in Chronic Diseases, Universidad Peruana Cayetano Heredia, Lima, Peru; 7 Melbourne School of Population and Global Health, University of Melbourne, Melbourne, Victoria Australia; 8 Institute for Global Health, University College London, London, UK; 9 Department of Medicine, School of Clinical Sciences at Monash Health, Monash University, Melbourne, Victoria Australia; 10 The George Institute for Global Health, University of New South Wales, Hyderabad, India; 11 The George Institute for Global Health, UNSW, Sydney, Australia; 12 The University of Sydney, Sydney, Australia; 13 Department of Public Health, Institute of Tropical Medicine, Antwerp, Belgium; 14 Department of Primary and Interdisciplinary Care, University of Antwerp, Antwerp, Belgium; 15 New York University School of Medicine, New York, USA; 16 Faculty of Health Sciences, Queens University, Kingston, Ontario Canada; 17 New York University College of Global Public Health, New York, USA

**Keywords:** Process evaluation, Implementation science, Complex interventions, Mixed-methods, Low and middle-income countries, Hypertension

## Abstract

**Background:**

Process evaluation is increasingly recognized as an important component of effective implementation research and yet, there has been surprisingly little work to understand what constitutes best practice. Researchers use different methodologies describing causal pathways and understanding barriers and facilitators to implementation of interventions in diverse contexts and settings. We report on challenges and lessons learned from undertaking process evaluation of seven hypertension intervention trials funded through the Global Alliance of Chronic Diseases (GACD).

**Methods:**

Preliminary data collected from the GACD hypertension teams in 2015 were used to inform a template for data collection. Case study themes included: (1) description of the intervention, (2) objectives of the process evaluation, (3) methods including theoretical basis, (4) main findings of the study and the process evaluation, (5) implications for the project, policy and research practice and (6) lessons for future process evaluations. The information was summarized and reported descriptively and narratively and key lessons were identified.

**Results:**

The case studies were from low- and middle-income countries and Indigenous communities in Canada. They were implementation research projects with intervention arm. Six theoretical approaches were used but most comprised of mixed-methods approaches. Each of the process evaluations generated findings on whether interventions were implemented with fidelity, the extent of capacity building, contextual factors and the extent to which relationships between researchers and community impacted on intervention implementation. The most important learning was that although process evaluation is time consuming, it enhances understanding of factors affecting implementation of complex interventions. The research highlighted the need to initiate process evaluations early on in the project, to help guide design of the intervention; and the importance of effective communication between researchers responsible for trial implementation, process evaluation and outcome evaluation.

**Conclusion:**

This research demonstrates the important role of process evaluation in understanding implementation process of complex interventions. This can help to highlight a broad range of system requirements such as new policies and capacity building to support implementation. Process evaluation is crucial in understanding contextual factors that may impact intervention implementation which is important in considering whether or not the intervention can be translated to other contexts.

**Electronic supplementary material:**

The online version of this article (10.1186/s12889-019-7261-8) contains supplementary material, which is available to authorized users.

## Background

A substantial challenge faced by implementation researchers is to understand if, why and how an intervention has worked in a real world context, and to explain how research that has demonstrated effectiveness in one context may or may not be effective in another context or setting [1]. Process evaluation provides a process by which researchers can explain the outcomes resulting from complex interventions that often have nonlinear implementation processes. Most trials are designed to evaluate whether an intervention is effective in relation to one, or more, easily measureable outcome indicator (e.g. blood pressure). Process evaluations provide additional information on the implementation process, how different structures and resources were used, the role, participation and reasoning of different actors [2, 3], contextual factors, and how all these might have impacted the outcomes [4].

Several authors have argued that in complex interventions, process measures used to examine the success of the implementation strategy, must be separated from outcomes that assess the success of the intervention itself [5]. The recent Standards of Reporting Implementation Studies (StarRI) statement consolidates and supports this concept [6, 7]. Given the significance of the causal pathway of a research intervention, which is crucial to future policy and program decisions, it is helpful to understand how different research programs, in different settings, employ process evaluation and the lessons that emerge from these approaches.

Since 2009, the Global Alliance for Chronic Diseases (GACD) has facilitated the funding and global collaboration of 49 innovative research projects for the prevention and management of chronic non-communicable diseases [8] with funding from GACD member agencies.^1^ Process evaluation has increased in importance and is now an explicit criterion for project funding through this program. The objective of this paper is therefore to describe the different process evaluation approaches used in the first round of GACD projects related to hypertension, and to document the findings and lessons learned in various global settings.

## Methods

### Data collection

Preliminary data on how the projects planned their process evaluations, were collected from the 15 hypertension research teams in the network in 2015 and was used to develop a data collection tool. This tool was then used to collate case study information from seven projects based on the following themes: (1) description of the intervention, (2) objectives of the process evaluation, (3) approach (including theoretical basis, main sources of data and analysis methods), (4) main findings of the study and the process evaluation, (5) implications for the project, policy and research practice and (6) lessons for future process evaluations. The case study approach recognizes that projects were at different stages of intervention/ evaluation. Each process evaluation was nested within an intervention study that was either completed or nearly completed.

### Data analysis

Information relevant to each of the agreed themes was documented by FL and JW using a data extraction sheet (see Table 1). The information was summarized and reported descriptively and narratively in relation to the themes above. Overarching issues were identified by the working group that had been established to oversee the project. The working group comprised researchers who had all been involved in the different process evaluations and helped to draw out the main implications of the process evaluation with respect to project and policy, as well as lessons to inform future process evaluations.

**Table 1:** Process evaluation approaches, implications and lessons from seven case studies

Study and Country	Intervention/ target population	Process Evaluation objectives and theoretical approach	Methods/ data sources	Main findings	Implications for project and policy	Lessons for process evaluation
Cost effectiveness of salt reduction programs in the Pacific Islands (Fiji and Samoa)	Government policy initiatives to reduce salt in foods and meals and community mobilization for behavior change to reduce salt intake.	Understanding reach, fidelity, dose, context, feasibility and lessons for future programs	Mixed methods: Interviews, collection of routine monitoring data, sub-analysis of population salt survey data	Trial outcome: No statistically significant reductions in mean salt intake. Increased awareness of negative impact of salt and some improvements in salt-use behavior.	1. Complex policy interventions need more time and clear implementation strategies 2. Multi-sectoral interventions require strong government leadership	1. Challenging and time consuming to collect and analyse data but adds to learning 2. Stakeholder interviews and measures of the implementation process should be done throughout the intervention period to inform necessary adaptations 3. Using mixed methods and several data sources allows for cross-checking and triangulation to enhance the validity of process evaluation data
A pre-post study design.		Approach: MRC process evaluation framework				
Project (Fiji): August 2012 –August 2016 Publication: January 2018 Project (Samoa): (January 2013 – December 2015), Publication: (August 2018)	Setting: Population wide.			Process evaluation: Intervention not implemented with full fidelity within timescale due to contextual factors. However, research capacityincreased, salt reduction mainstreamed in government policies and mechanisms for engaging industry established.		
Treating hypertension in rural South Africa: A clinic-based lay health worker to enhance integrated chronic care in Mpumalanga South Africa A cluster randomized controlled trial.	Lay health workers (LHW) improving management of hypertension by undertaking simple tasks and freeing nurses to focus on clinical work.	Understanding process of developing the intervention, implementation context and mechanisms and processes that led to changes in patient outcomes	Mixed methods: Observations, focus group discussions, interviews, diaries	Trial outcome: Population control of hypertension did not improve	1. Strong management, skilled LHW, functional equipment and good relations, are essential for success in task shifting	1. Realist evaluations can complement and be combined with randomized controlled trials. 2. Study sites that are within the same geographical area can be diverse in context.
				Process evaluation: LHW Intervention made clinics function better and increased patients’ adherence to appointment. Health system factors affected implementation.		
Project (April 2013 – December 2015 Publication: (November 2017)	Setting: rural clinics	Approach: Realist evaluation				
Diagnosing hypertension—Engaging Action and Management in Getting Lower BP in Indigenous communities in Canada and rural communities in Tanzania)	Health care SMS text messages supporting patient hypertension self-management and facilitating decision support for health care providers.	To assess the major active components of the intervention, technology of the intervention, task shifting, cultural congruence and unintended consequences as part of formative research to inform the intervention strategies	Mixed methods: Research notes for implementation. Reflective discussion sessions with researchers and community, interviews and focus groups	Trial outcomes: Ongoing	1. Important to prepare intervention using local knowledge 2. Need to establish ongoing dialogue between community and researchers 3. Identify strengths and challenges for implementation.	1. Community Based Participatory Research values local knowledge, cultural understanding 2. Formative research should be part of process evaluation in trials.
				Formative research: Showed discrepancies between text messages created by researchers and how recipients felt about them. This informed development of text messages congruent to population’s motivation for behavior change.		
A prospective randomized trial.	Setting: indigenous communities in Canada and rural communities in Tanzania					
Project (January 2012 – January 2017) Publication: (April 2017)		Approach: Community based participatory research				
Optimizing linkage and retention to hypertension care to reduce blood pressure in rural western Kenya - Kosirai and Turbo A cluster randomized controlled trial Project (April 2012 – March 2017) Publication: (January 2016)	Community Health Workers (CHW) equipped with a tailored behavioral communication strategy and smartphone technology to increase linkage and retention of people with hypertension	Determining fidelity, CHW knowledge, skill retention, attitudes, patient perceptions and barriers to care linkage and retention.	Mixed methods: Survey, focus group discussions, collection of process indicators, written tests and clinical examinations.	Trial outcomes: Study completed, but analyses still underway.	1. Continuing training, support, and surveillance necessary for program fidelity	1. Multi-modality approach (qualitative and quantitative; combination of observations, discussions, and testing) is critical in complex intervention 2. Process evaluation should start earlier 3. Mixed methods captures areas of data discordance and concordance
				Process Evaluation: Low implementation fidelity among CHWs. Initial CHW training significant in some areas and not others. Skills retention among CHWs was sub-optimal		
	Setting: rural communities in Kenya	Approach: RE-AIM framework and Realist evaluation				
Salt substitute to reduce blood pressure at the population level in northern Peru A stepped wedge trial Project (March 2012 – March 2017) Publication: (July 2017)	Replacing high sodium salt for a salt substitute (low-sodium, high-potassium salt) to reduce blood pressure (BP) among those 18 years and over.	To understand strategies for incorporating salt substitute, barriers and facilitators to introducing salt substitute and perceived health impact for salt substitute	In-depth interviews	Trial outcomes: Population-level BP was reduced,	1. Acceptance of a new product was gradual 2. Important to target those preparing meals 3. Acceptance was increased by providing a substitute for salt without financial cost.	1. Process evaluation data to be collected repeatedly, at the beginning and throughout the implementation time
				Process evaluation: Good relations between community and researchers facilitated uptake of the salt substitute. Women did not consult family members when they introduced salt substitute		
	Setting: six villages	Approach: Phenomenology: understanding study participants’ and other stakeholders’ perception				
Improving control of hypertension in rural India: Overcoming barriers to diagnosis and effective treatment A cluster randomized controlled feasibility trial Project (January 2014 – October 2016), Publication (May 2018)	Training Accredited Social Health Activists (ASHAs) to support patients with hypertension to adopt self-management behaviours and improve BP control	Assessing fidelity and factors that might have impacted on the ASHA training outcomes	Mixed methods: Interviews, focus group discussions, intervention meeting reports.	Trial outcomes: Study completed, but analyses still underway.	1. Need for culturally appropriate training materials for NCDs 2. Importance of adequate interactive and innovative training, retraining, and supervision 3. Need for timely remuneration and incentives for ASHAs	1. Mixed methods gave an in-depth understanding of the intervention 2. Difficult to collect data in 3 different cultural sites using the same methods
				Process evaluation: ASHA training was feasible and led to changes in knowledge, skills, and motivation. ASHAs delivered the intervention appropriately. Community appreciated their roles		
	Setting: 3 regions in rural India	Approach: Kirkpatricks’ four level evaluation model				
Early use of low-dose triple combination of BP lowering drugs in improving BP control in Sri Lanka A randomized controlled trial • Project: (February 2014–December 2017), Publication: August 2018	Simplified treatment regimen of a half -dose, three-in-one blood pressure lowering pill (Triple pill) for improving hypertension control	Investigating factors and their interplay behind the results, patients and providers experience and barriers and facilitators to implementation.	Semi-structured interviews	Trial outcomes: More patients in the intervention arm reached their blood pressure targets compared to usual care.	1. Training of General Practitioners for the management of hypertension including combination therapy 2. Ensuring the availability of combination therapy	1. Process evaluation data collected before trial results are available, helps in exploring views uninfluenced by trial results.
	Setting: patients with hypertension in outpatient departments in 11 urban hospitals	Approach: Framework analysis method		Process evaluation: Patients and providers liked the triple pill because of its ease of use (single pill, once a day dosing) and significant BP control. No major safety issues were reported.		

## Results

### Countries and interventions studied

The seven process evaluations were from low- and middle-income countries (LMICs) that had either completed or nearly completed their process evaluations including Fiji and Samoa, South Africa, Kenya, Peru, India, Sri Lanka, Tanzania and indigenous communities in Canada, (Table 1). The countries referred to in this manuscript were countries in which projects were a) funded through the GACD and b) contained process evaluations at a sufficiently advanced stage to include in the analysis. These process evaluations were part of pragmatic trials of innovative interventions to prevent and manage hypertension in the areas of salt reduction, task redistribution, mHealth, community engagement and blood pressure control [8]. Although the studies generally took place in LMICs, there were varying geographical, cultural and economic settings within and across countries (Table 1). Most of the interventions (five) were tested in randomized controlled trials with one stepped wedged trial and one pre-post study design. The duration of the studies was three to 5 years (Additional file 1).

### Objectives and theoretical approaches to process evaluations

The specific objectives for each process evaluation were tailored to the broader objectives for each project and however, they were generally aimed at understanding factors that would have affected the implementation process and the impact of this process on trial outcomes. These objectives were achieved through the collection of qualitative information about context, mechanisms, feasibility, acceptability and sustainability of the interventions and quantitative information about fidelity (extent to which the intervention is implemented as intended), dose (how many units of each intervention are delivered) and reach (extent of participation of the target population) [2].Two process evaluations (in Peru and Sri Lanka) were limited to assessing barriers and facilitators which affected implementation, and in Peru the project had benefited from its previous formative research [9]. In India, the process evaluation had only evaluated the ASHA training and not the entire intervention. For the DREAM GLOBAL in Tanzania and Canada, the investigators have so far undertaken and reported on formative research as part of its process evaluation and have published their process evaluation framework protocol [7]. This formative research is aimed at assessing the major components of the intervention and how these components should vary among and between people, countries and cultures.

The theoretical approaches to process evaluation differed in the seven case studies, including use of the United Kingdom’s Medical Research Council (MRC) framework for process evaluation (Fiji/Samoa) [2, 10], the realist evaluation approach (South Africa and Kenya) [11], community-based participatory evaluation theory (Tanzania and Canada) [7], the RE-AIM framework (Kenya) [12], Kirkpatricks’ four level evaluation model (India) [13], phenomenology (Peru) [14] and Framework analysis method (Sri Lanka) [15] (see Table 2).

**Table 2:** Description of the different theoretical approaches used in the GACD process evaluations

Theoretical approach	Description
MRC Process Evaluation Framework	The MRC Process Evaluation Framework is designed for complex interventions. The framework explains that outcomes in an intervention are a result of configuration of implementation (structures, resources, and processes), context (internal and external social, cultural and economic factors) and mechanisms (reasoning among program participants) [2, 10].
Realist Evaluation	The Realist Approach to process evaluation is based on Pawson and Tilley’s realist thinking that answers the questions “what works for whom, under what conditions and how” [10, 16, 17]. Realist evaluation examines underlying mechanisms (participants’ reasoning and how they interacted with the intervention) and how they impacted on the outcomes in different contexts [11].
Community Based Participatory Evaluation Theory	In Community Based Participatory Evaluation theory, researchers argue that participatory evaluation theory is an ideal framework for process evaluations when trials are implemented in multiple cultural settings [7]. A constructivist approach is incorporated where individuals and communities participate in focus groups to give their views and lived experiences of the intervention based on the evaluation framework [7].
RE-AIM Framework	The RE-AIM framework was developed to measure how interventions that have proved effective in one area, can be expanded to a wider scale in multiple areas [12]. The framework offers a standardized framework of five dimensions: Reach (participation of target population), Efficacy (effects of the program), Adoption (uptake of the intervention), and Implementation (extent to which the intervention is implemented as intended) and Maintenance (sustainability of the intervention’s benefits) [12].
Four Level Evaluation Model	The ‘four level’ evaluation model for training programs by Kirkpatrick & Kirkpatrick, presents different levels for evaluating training programs. The levels include: the degree to which participants react favorably, the degree to which participants acquire the intended knowledge and skills, the degree to which participants apply what they have learned and the degree to which targeted outcomes occur [13].
Phenomenology	Phenomenological research, drawn from anthropology and social sciences, describes “lived experience” and people’s perspective on a given issue (or phenomenon) and their interpretation thereof. This usually involves qualitative analysis of narrative data [14].
Framework Analysis	The Framework analysis method developed by Jane Ritchie and Liz Spencer is suitable for thematic analysis of qualitative and textual data. The approach provides a step-by-step method of structuring the data in a matrix to compare and contrast data. The steps include: transcription, familiarization with the data, coding, developing a working analytical framework, applying the analytical framework, charting data into the framework matrix and Interpreting the data [15].

### Main sources of data and data collection methods

Most of the investigators used a mixed methods approach in their data collection and inquiry, integrating quantitative and qualitative data within a single study [18]. For all seven case studies, qualitative data were obtained using one or more of the following methods: interviews, observations, focus group discussions, implementer diaries, researcher notes and reports of meetings. Quantitative data were obtained from surveys, metrics of process measures, written tests and clinical examinations. In most of these cases, data were collected and analyzed by researchers who were members of the study team but were adept at analyzing these types of data and so were specifically employed to conduct process evaluation. In two studies, phenomenological and framework analysis approaches were applied with only qualitative data used in their evaluation. This was because their process evaluation solely focused on assessing barriers and facilitators.

### Main findings of process evaluations

Although the studies reported in this paper had very distinct interventions and outcomes, there were similarities in the findings of the process evaluations across the studies. The process evaluations of the salt reduction interventions in Fiji and Samoa demonstrated that the absence of a significant reduction in salt, in either country, could be explained by the fact that the interventions were not implemented with full fidelity (i.e. implemented in line with the study protocol). This was partly due to contextual factors including political and management changes, a cyclone in Fiji (affecting normal diets) – and partly due to lack of time for the intervention to take effect. However, the process evaluation also highlighted the fact that the projects had resulted in increased research capacity among government and research institutions that participated in the study. In addition, new government policies for salt were being integrated into the new Food and Nutrition security strategy in Fiji, while in Samoa government proposals for taxation of packaged foods high in sugar and salt, were being considered.

In South Africa, although control of hypertension was not improved, the lay health worker (LHW) intervention enabled the functioning of clinics to be streamlined, partly by improving the appointment system for chronic patients. The process evaluation enabled investigators to conclude that shifting certain medically and socially oriented tasks from nurses to LHWs could relieve the burden on nurses, improve delivery of chronic care, and improve functioning of primary care clinics. However, there was non-linearity between implementation process and outcomes at clinic level. The variability in implementation and outcomes between sites were likely a consequence of different levels of patient load and resources, nature of relationships and clinic management [19, 20]. For instance, in clinics with high patient loads LHWs were unable to complete all their tasks.

The process evaluation in Tanzania and Canada contributed to the formative stage of the intervention by identifying discrepancies between text messages created by researchers and those preferred by recipients, thereby enabling a change in the study design prior to commencement.

In Kenya, although analysis of the main trial outcomes had not been completed at the time of developing this paper, process evaluation data showed low fidelity in implementing certain components of the intervention, sub-optimal retention of skills among community health workers (CHWs), and gaps in recall of training elements by CHWs in some topics. Initial training of CHWs was more effective to help CHWs recognize complications, non-pharmacologic treatments and causes of hypertension, than to recognize the signs and symptoms of hypertension and the possible side effects of medication. In response to these findings, the project subsequently incorporated training that encompassed these other issues.

The salt substitute intervention in Peru was effective in reducing population levels of uncontrolled BP. The process evaluation enabled the investigators to determine that effective implementation of the intervention was attributable to (1) good relations and trust between researchers and the community facilitating the launching of the trial in the area, and thus take advantage of this established engagement platform aided the intervention’s uptake through trust; and (2) targeting of women during the intervention as the critical primary receptors of the intervention due to their role as food preparers in homes.

The process evaluation in India is still underway, but the process evaluation of the training of Accredited Social Health Activists (ASHAs) demonstrated the intervention was successfully implemented by the ASHA which improved skills, knowledge and motivation among the ASHAs [21, 22].

In Sri Lanka, patients and providers liked the triple pill because of its ease of use (single pill, once a day dosing) and significant BP control. At the beginning of the trial, providers expressed apprehension about initiating treatment with the triple pill in treatment naïve hypertensive patients. Over time, they became comfortable as no major safety issues were reported and the extent of BP lowering achieved was substantial. Providers expressed a willingness to prescribe the triple pill and patients were willing to use it if it was made available after the trial.

These findings from the seven cases demonstrate the role of process evaluations in describing the implementation process despite variation in study outcomes.

### Implications for the projects and policy

A series of implications for projects and policies were highlighted by the process evaluations. For salt reduction interventions, these included the need for adequate time between baseline and follow up for the implementation to take effect; the need for strong leadership (diverse, experienced and representative) and clear roles for multi-sector advisory bodies; regular communication with stakeholders; and consideration of consumer acceptability and affordability of salt-reduced products. For the LHW intervention, the importance of a supportive and well-resourced clinic environment, strong management of the Primary Health Care facilities, and motivated staff that relate well to the patients were identified as fundamental for successful task shifting operations. The importance of continued training, communication and programmatic support was highlighted through a number of process evaluations. The process evaluation in the CHW study in Kenya showed the need for additional education about signs and symptoms of hypertension and treatment side effects as well as intensive, repeated training regarding hypertension management. Similarly, findings from the ASHA program in India emphasized the need for culturally appropriate training materials, delivered using interactive and innovative methods. It also showed the need to align project tasks and responsibilities with CHW incentives as without this, their morale for work would decline. Implementing a low dose, combination treatment strategy in Sri Lanka highlighted the need for education and training for prescribing physicians around the benefits of early use of combination therapy for treatment of hypertension and ensuring availability of the combined therapy for hypertension.

## Discussion

This paper provides an overview of the application and findings of process evaluations in different hypertension implementation research projects in various LMICs and Indigenous communities in Canada. The major objective of each process evaluation was to understand the implementation of interventions. The lessons from this paper relate to: 1) the feasibility and application of process evaluations; and 2) the relevance of process evaluation results for understanding implementation effects and their impact. Research teams used a variety of frameworks and methods, each deemed to be appropriate and feasible for the research team and context. Despite the variety of methodological approaches, with some differences and overlaps, most process evaluations shared similar goals of describing the processes, structures and resources of the respective studies.

Our study has demonstrated the need to consider process evaluation early in the research cycle so as to optimize design and data collection throughout the implementation cycle. When done early in the project cycle, process evaluations can help to optimize implementation of the intervention, as was done in Kenya through repeated training for health providers delivering the intervention. In many projects, such as in the South African study, the relationship between intervention and outcome included pathways within the clinics that differed to those originally hypothesized. In addition, process evaluations allowed for documenting unexpected results. The process evaluation of the salt reduction programs in the Pacific Islands [23, 24], pointed to natural and political context slowing down the implementation of the intervention, but also demonstrated how the project led to new mechanisms for intersectoral collaboration. In general, the process evaluations illuminated that maintaining full fidelity to the original implementation plan is often difficult to achieve, with resource constraints further affecting the implementation process. These findings are vital in explaining and understanding the context in which trial outcomes were (or were not) achieved.

Some of the findings from this study align with findings from other authors which are quoted in this paragraph. The causal relationship between implementation and outcome is, in real life implementation, affected by the adaptability (or unpredictability) of actors, and the wide range of influencing elements [25] including geographical and community setup. Using a mixed-methods approach deepens the understanding by providing different perspectives, validation and triangulation by using multiple sources [2, 26]. Qualitative analysis enables exploration of the acceptability of an intervention, how it worked and why [27]. Quantitative analysis are important to measure elements of fidelity [27]. This implies the need for a comprehensive skill set within the research team.

The strength of this report is that it is based on a wide range of research projects and applications of process evaluations in different LMICs but focused on one chronic disease; hypertension. The selection of study cases, being only hypertension projects funded by GACD, was limited by the study set-up and timing of documentation. This contributes to a broad insight of how process evaluation can be incorporated into studies and used in different interventions and settings. The detailed case studies compiled by teams, coupled with the regular GACD overarching working group meetings allowed the experiences of process evaluations in different phases of the implementation process to be documented. Other GACD funded projects, for instance on diabetes, had not yet progressed far enough in their work to be able to contribute findings. The choice for including in-depth case study analyses necessitates a level of trust between the authors and the research teams, especially since most teams have not yet finalized their analyses nor published their findings. We chose not to include other GACD projects, thereby reducing the scope of projects that could contribute to the analyses. We believe the current approach facilitated more in-depth analyses, thereby enriching the findings of this study.

The implications of this study pertain to the discipline of implementation research and to the engagement with implementers and decision-makers. All of the frameworks adopted provided useful outcomes. The choice of a framework and method should be guided by the key questions that need to be answered to understand the implementation process and the skills and preferred methodological approaches of the researchers.

For process evaluations to be informative, we need a diverse skill set. Project management data are required to inform fidelity of the implementation. Further analysis of observations and interviews with people involved in the intervention is required to gain field-based understanding of the evolution of the intervention, the mechanisms triggering effect and the perceptions of actors on what crucial elements or moments have been in the evolution. Thus, the evaluations must be interdisciplinary, combining techniques and methods from a range of sources including project management, anthropology, psychology and clinical sciences.

The findings of process evaluations are crucial to understand the pathways between intervention and impact, so as to optimize implementation, impact and inform scalability of the interventions. This requires planning from the project outset, and engagement with implementers and decision makers throughout program implementation [1]. This is contrary to the classical set-up of most trials in which deviation from the study protocol is not acceptable because it interferes with the evaluation of effectiveness. The choice about whether to involve implementers in the process evaluation depends on the design of the primary study. The aim of many implementation studies is to test effectiveness instead of efficacy, and this requires more flexible study designs, such as an adaptive trial design, enabling optimization of the implementation throughout the course of the project. This requires ongoing dialogue between implementers and researchers evaluating this process. In studies where this interference is deemed problematic, researchers can opt for a more distant relationship between the intervention and evaluation teams, such as that occurring in the study of hypertension treatment in South Africa (case study 1.2).

The findings from most process evaluations demonstrate both the importance and the challenges of adapting initial research plans to accommodate the constraints in a (low resource) context. Detailed discussions are required to understand context and expectations of local stakeholders. This necessitates formative research and establishment of trusting relationship to shape mutual commitment to action between researchers and local communities. This has implications for the design of research. Many research projects experience delay in the formative and implementation phases of their projects. Only three process evaluations have so far been published [21, 23, 24]. This points to the need to reflect on planning and funding of research cycles.

### Lessons and recommendations for process evaluations

**Fig. 1:**
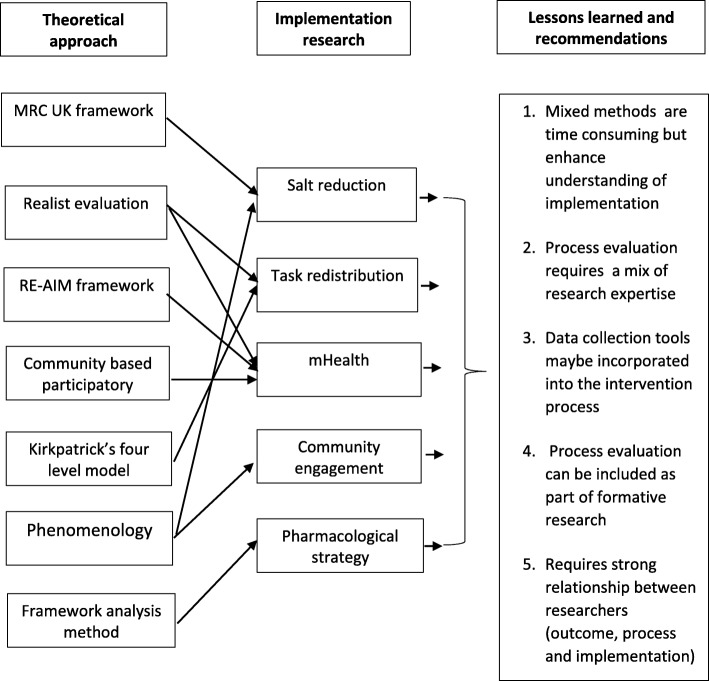
Lessons learned from the process evaluations

A range of lessons for process evaluation as part of implementation research in LMIC have been identified through this study (Fig. 1). A common theme that emerged was that while mixed methods approaches can be time consuming and generate a vast amount of data, they significantly enhance understanding of the implementation of complex interventions as well as generate a wealth of learning to inform future projects. For instance, the semi-structured interviews used in the Fiji and Samoa process evaluation, produced qualitative data, i.e. experiences of government institutions, the food industry and other stakeholders. This information helped to explain that the lack of intervention effect on salt intake was likely at least partially attributable to the short intervention duration and the fact that policy changes had yet to take effect.

Incorporating process evaluation data collection tools into the intervention process from the onset was identified as crucial for process evaluation. Process evaluations cannot be conducted retrospectively as investigators cannot go back and collect the required data. For instance, for the clinic based LHW intervention in South Africa, it was ideal to observe and understand how nurses interacted with the intervention as it was being implemented. Thus, process evaluations should be fully embedded into the intervention protocol or a separate process evaluation protocol should be developed alongside the intervention protocol. In Sri Lanka, collecting process evaluation data before the study outcome data were available helped in exploring implementation processes without unintentionally influencing investigator or patient behavior in the study.

Some investigators who commenced their process evaluation after the intervention had begun, e.g. the CHW project in Kenya, felt that it may have been helpful to have started this earlier in the study life-cycle. Other investigators who incorporated process evaluation in formative research and situation analyses reported that this approach helped identify which specific process measures should be collected during the intervention. One study team deliberately did not collect process evaluation data until after the study was complete so as to not affect investigator or patient behavior in the study itself. Therefore multiple considerations should be taken into account when designing process evaluations.

Experiences from six of the projects support the MRC recommendation for strong relationship and consultations between researchers responsible for the design and implementation of the trial, outcome evaluation and process evaluation [2]. However, whilst the teams reported positively on coming together to exchange experiences at different stages of the project, it was felt that additional interim assessments of process throughout the project would have further strengthened implementation of the interventions. In some teams, discussion of the preliminary results of the process evaluation by the broader project group, including local country teams, was an essential part of the data synthesis and greatly enhanced the validity of the results by clarifying areas in which the researchers might not have understood the data correctly.

The DREAM GOBAL process evaluation demonstrated the need for formative research that informed the mHealth projects for rural communities in Tanzania and Indigenous people in Canada as well as the value of using a participatory research tool [28]. This tool helped to identify: a) key domains required for ongoing dialogue between the community and the research team and b) existing strengths and areas requiring further development for effective implementation. Applying this approach, it was found that key factors of this project, such as technology and task shifting required study at the patient, provider, community, organization, and health systems/setting level for effective implementation [7].

## Conclusion

The analysis of process evaluations across various NCD-related research projects has deepened the knowledge of the different theoretical approaches to process evaluation, the applications and the effects of including process evaluations in implementation research, especially in LMICs. Our findings provide evidence that, whilst time-consuming, process evaluations in low resource settings are feasible and crucial for understanding the extent to which interventions are being implemented as planned, the contextual factors influencing implementation and the critical resources needed to create change. It is, therefore, essential to allocate sufficient time and resources to process evaluations, throughout the lifetime of these implementation research projects.

## Additional files

Additional file 1: Annexures (case studies) (DOCX 39 kb)

## Data Availability

The datasets generated during and/or analysed during the study are available from the corresponding author on reasonable request.

## Abbreviations

ASHA: Accredited social health activist
CHW: Community health worker
FGD: Focus group discussion
GACD: Global alliance for chronic diseases
LHW: Lay health worker
LMICs: Low and middle-income countries
MRC: Medical research council
NCD: Non-communicable disease
RE-AIM: Reach, efficacy, adoption, implementation, maintenance
SMS: Short message service
StarRi: Standards for reporting implementation studies

## Footnotes

1 Ministry of Science, Technology and Productive Innovation (Argentina), National Health and Medical Research Council (Australia), São Paulo Research Foundation (Brazil), Canadian Institutes of Health Research, Chinese Academy of Medical Sciences, Research & Innovation DG (European Commission), Indian Council of Medical Research, Agency for Medical Research and Development (Japan), The National Institute of Medical Science and Nutrition Salvador Zubiran (Mexico), Health Research Council (New Zealand), South African Medical Research Council, Health Systems Research Institute (Thailand), UK Medical Research Council and US National Institutes of Health
