# Supplementary material for: Process evaluation in the field: global learnings from seven implementation research hypertension projects in low-and middle-income countries
Source: BMC Public Health. 2019 Jul 16;19:953. doi: 10.1186/s12889-019-7261-8 (PMC6651979; doi:10.1186/s12889-019-7261-8)
Supplement: Supplementary file 1 — Annexures (case studies) (DOCX 39 kb) [file 12889_2019_7261_MOESM1_ESM.docx]

**Annexure**

**Annex 1: Case studies of the seven hypertension teams**

1. **Cost effectiveness of salt reduction programs in the Pacific Islands**

Project dates (Fiji): (August 2012 –August 2016); Publication dates: (January 2018)

Project dates (Samoa): (January 2013 – December 2015); Publication dates: (August 2018)

*Theoretical Approach: Medical Research Council’s Framework for Process Evaluation*

The MRC framework for evaluating complex trials explains how outcomes are a result of the configuration of implementation, e.g. structures, resources, and processes, context, e.g. internal and external social, cultural and economic factors, and mechanisms (reasoning among program participants) ^1-3^. We used MRC’s framework for complex interventions to evaluate the impact of multi-faceted interventions to reduce population salt intake in Fiji and Samoa ^4^. The process evaluation was aimed at generating implementation process data to assess the extent to which the interventions were implemented as planned and understand factors that may have impacted program outcomes among different groups.

We collected qualitative and quantitative data through a mixed method approach. The data helped in defining and understanding the causal assumptions underlying the interventions described through a logic model and answered the following questions: (a) were the program interventions delivered with high fidelity, dose and reach? (b) Was the multi-faceted intervention program a feasible, acceptable and sustainable model for this context? (c) How did context affect implementation? (d) How can the salt reduction intervention be improved for replication in other countries?

Although there was no reduction in salt intake in Samoa, and the reduction in Fiji was not statistically significant, the process evaluation allowed for increased understanding of the contextual factors underlying the intervention implementation. These included the impact of natural disasters (cyclones), political influence, and staff and governance changes on program delivery. The project raised consumer awareness of the negative impact of salt on health in both countries, strengthened cross-sectoral collaboration on NCDs, and established a new mechanism for engaging with the food industry. Salt reduction is now mainstreamed into government policies including through voluntary or regulatory salt standards and salt education as part of national NCD or nutrition strategies. Workforce capacity to address NCDs has been increased in both countries and longer term monitoring of impacts of sustained programs is planned through future WHO STEPS surveys in 2018/19.

The process evaluation has raised lessons for salt reduction programs. These include the need for more time to ensure that interventions can take effect; the importance of clarifying and strengthening the roles of the multi-sector advisory groups and maintaining momentum through regular communication with stakeholders; the need to establish clear mechanisms for policy implementation and monitoring and the importance of more strategic communication approaches to behavior change. These lessons are being used to inform ongoing salt reduction programs in Fiji and Samoa but are equally applicable to other parts of the world.

1. **Treating hypertension in rural South Africa: A clinic-based lay health worker to enhance community-based outreach services for integrated chronic care**

Project dates (April 2013 – December 2015); Publication dates: (November 2017)

*Theoretical Approach: Realist Evaluation Approach*

Based on Pawson and Tilley’s realist approach, realist evaluations answer the question “what works for whom, under what conditions and how” ^3 5 6^. Realists examine mechanisms underlying change (participants’ reasoning and how they interacted with the intervention) and how they impacted on the outcomes in different contexts ^7^. We tested whether providing two lay health workers (LHW), working alongside the nurses in the clinics, could improve the management of hypertension in rural South Africa ^8^. Several tasks were redistributed from nurses to LHWs (intervention) which included booking patients for their appointment and measuring blood pressure. A detailed realist evaluation aimed at understanding under what context and through what mechanisms, the clinic based LHW intervention might enhance integrated chronic care for hypertensive patients and modify patient outcomes.

The evaluation explored the intervention and operation of the clinics throughout the trial implementation period using both qualitative (FGDs, Interviews, observations, diaries) and quantitative data (Patient exit interviews, clinic data). The main evaluation questions included (a) How did the intervention development process affect the functioning of the LHWs for each clinic? (b) How did the different clinic contexts affect the implementation of the intervention? (c) How did the different mechanisms and broader health system factors affect implementation of the intervention? (d) What processes led to change (or not) in the patient and related outcomes? We used a case study approach to compare and contrast experiences in eight case clinics (four randomized to intervention).

The intervention did not succeed in improving population control of hypertension but helped clinics to function better. Patients’ attendance on their appointment dates, management of the appointment system, filing system and prepacking of medication, improved. However, BP machines that did not work, erratic supply of drugs and materials, poor clinic management and poor working relations affected the implementation of the intervention. Clinics with good infrastructure, equipment, management, better nurse levels, low patient loads, had positive effects in the work of the LHWs. These were also clinics where staff related well among themselves and with patients, supported the work of LHWs and had motivated and skilled LHWs. Such clinics had positive clinic level outcomes that included patients adhering to their appointment dates and identifying patients with raised BP.

The process evaluation concluded that clinic based LHWs can relieve burdened nurses and improve delivery of chronic care and functioning of primary care clinics when certain tasks are shifted from nurses to LHWs. However, this success appears to be dependent on a supportive environment with functional equipment, consistent supply of resources, strong management of the PHC facilities, and motivated staff that work as a team and relate well to the patients. In the LHW intervention, variations in implementation and outcomes were likely a result of different levels of patient load and resources, nature of relationships and clinic management.

1. **Diagnosing hypertension—Engaging Action and Management in Getting Lower BP in Indigenous and LMIC (DREAM-GLOBAL)**

Project dates (January 2012 – January 2017); Publication dates: (April 2017)

*Theoretical Approach: Participatory Evaluation Theory*

Researchers argue that participatory evaluation theory is an ideal framework for process evaluations when trials are implemented in multi-cultural settings ^9^. A constructivist approach is incorporated where individuals and communities participate to contribute their views and lived experiences to the evaluation framework ^9^. DREAM-GLOBAL used mobile technology (mHealth) to improve hypertension management in rural Tanzania and indigenous communities in Canada. The intervention consisted of health care short message services (SMS) text messages to support patient hypertension self-management and to facilitate decision support for health care providers. A process evaluation of the trial documented active ingredients in everyday practice, how they exerted their effect and how these varied among and between people, countries and cultures, and any unanticipated effects of the intervention.

DREAM-GLOBAL used community-based participatory research methods and formative research data to develop a framework for the process evaluation that can be adapted to multiple geographic, cultural and policy settings. Formative research identified four human organizational levels of participants impacted by the mHealth intervention which included patients, providers, community and organizations actors and health systems/settings. Using qualitative and quantitative data, process evaluation questions explored the following five themes in each of the human organization levels: (a) the major active components of the intervention, (b) technology of the intervention, (c) cultural congruence, (d) task shifting, and (f) unintended consequences.

At the time of writing, findings from the process evaluation of this intervention were not yet ready. These results are from the formative research. In terms of text message development, they found that discrepancies may have developed between the evidence-based text message created by researchers and the message received by the recipients in mobile health interventions in diverse cultural settings. These discrepancies were rooted within six mediators of meaning: (a) negative or non-affirming framing of advocacies, (b) fear- or stress-inducing content, (c) oppressive or authoritarian content, (d) incongruity with cultural and traditional practices, (e) disconnect with the reality of the social determinants of health and the diversity of cultures within a population, and (f) lack of clarity and/or practicality of content. These mediators informed the development of the SMS messages for the intervention ^10^.

Community Based Participatory Research process utilized local knowledge to understand implementation issues. Process evaluation researchers developed a tool, the I-RREACH (Intervention and Research Readiness Engagement and Assessment of Community Health Care) tool to guide implementation of interventions in low-resource environments ^11^. Specifically, the tool helped to prepare for a process evaluation by identifying: (a) key domains required for ongoing dialogue between the community and the research team as well as (b) existing strengths and areas requiring further development for effective implementation.

1. **Optimizing Linkage and Retention to Hypertension Care in Rural Kenya (LARK study)**

Project dates (April 2012 – March 2017); Publication dates: (January 2016)

*Theoretical Approach: Reach, Effectiveness, Adoption, Implementation and Maintenance (RE-AIM) Framework*

The RE-AIM framework offers a standardized framework of five dimensions: **R**each (participation of the target population for the intervention), **E**fficacy (positive and negative effects of the program), **A**doption (uptake of the intervention in agencies and setting), **I**mplementation (extent to which the intervention is implemented as intended in the real world) and **M**aintenance (sustainability of the intervention’s benefits) that can be combined to determine overall public health impact ([14](#_3rdcrjn)). The LARK study used both a realist approach ^12^ and RE-AIM framework in its process evaluation. The trial aimed to determine whether community health workers (CHWs), equipped with a tailored behavioral communication strategy and smartphone technology, could increase linkage and retention of hypertensive individuals to a hypertension care program and reduce blood pressure among them ^13^.

The process evaluation was designed to assess: (a) implementation fidelity, (b) CHW knowledge and skill retention, (c) CHW attitudes toward intervention components, (d) CHW and patient perceptions of intervention components and continuing barriers to linkage and retention to care. Focus group discussions (FGDs) assessed **A**doption and **I**mplementation, summary statistics of process measures captured the **R**each of the LARK trial, a usability survey about the paper-based and smartphone decision-support tool assessed the possibility of **M**aintenance of the decision-support tool portion of the LARK protocol, a self-administered written test of hypertension knowledge (written test) and objective structured clinical examinations (OSCEs) assessed **E**ffectiveness and **I**mplementation categories of the RE-AIM framework.

CHWs exhibited relatively low levels of study intervention fidelity including use of decision-support tools. Male CHWs were better at counseling than female CHWs. Fidelity to behavioral assessment use was highest among smartphone CHWs compared to usual care CHWs. The initial CHW training was effective in teaching CHWs to recognize complications of hypertension, non-pharmacologic treatments for hypertension and causes of hypertension. Initial CHW training was not effective in training CHWs to recognize signs and symptoms of hypertension and the possible side effects of medication. OSCEs showed that initial CHW training effectively taught CHWs when to refer to higher level of care for hypertensive patients. All other domains tested with the OSCEs showed sub-optimal skill retention and called into question the effectiveness of initial CHW training.

Continuing training and programmatic support was necessary to optimize fidelity of interventions. Additional education about signs and symptoms of hypertension and treatment side effects appeared necessary for all CHWs as well as intensive, repeated training regarding hypertension management. The OSCE was a critical component of process evaluation for its ability to reveal fidelity gaps in study protocol. The written test revealed knowledge deficits that affected study protocol implementation. FGDs assisted with understanding the context around protocol implementation or lack thereof.

1. **Salt substitute to reduce blood pressure at the population level in northern Peru**

Project dates (March 2012 – March 2017); Publication dates: (July 2017)

*Theoretical Approach: Phenomenology*

In Peru, a salt reduction project used a stepped wedge trial design to implement a population-level intervention to replace high-sodium salt for a salt substitute (low-sodium, high-potassium salt) to reduce blood pressure levels among adults aged 18 years and over in the semi-urban area of Tumbes ^14^. The implementation strategy involved a range of participants including villagers and their families (intervention targets) and local leaders, local institutions and local, regional and national authorities (key stakeholders) through a social marketing campaign. Process evaluation for the trial aimed at learning about the receptivity of the salt substitute by the local population, use of the salt substitute inside the household and the perceived effects of the use of the salt substitute.

A qualitative approach (phenomenology), drawn from anthropology and social sciences research, was used aiming at understanding the experiences of individuals in rural Tumbes using a salt substitute and the process of incorporating it into their everyday cooking practices. Data were collected using in-depth interviews with women heads of household and local promoters of the salt substitute. The research questions included: (a) what strategies did women used to incorporate the salt substitute in their everyday cooking and how did these change over time? (b) What were the main barriers to introduce the salt substitute? (c) What were the main facilitators for introducing the salt substitute? (d) What was the perceived health impact of using the salt substitute?

Women in the six rural villages did not consider it problematic to start using the salt substitute. One of the main reasons for this was the trust that villagers had with the local research center that was in charge of providing the salt substitute to the women in the villages. At first it took women some time to identify the amount of the salt substitute to use in order to get the desired taste. They used the salt substitute both for cooking and for salads, however women reported that when used in salads it was possible to detect a different “metallic” taste. Contrary to what the researchers expected regarding negotiations between women and family members to introduce the salt substitute, women stated that they did not consult anybody, they simply introduced it and if asked about the changes in the taste they stated that they were using this new product that was healthier than the “older salt”. At the beginning, participants had to swap their regular salt with the new salt substitute, and the project team provided packages of salt substitute replacements upon the request

Process evaluation showed that people in the target communities did use the salt substitute and after some time, families got used to the taste of this “new salt”. Thus, accepting a new product in a diet appears to be a gradual process. It was also important to target women or the people responsible for the preparation of the meals at home. For policy makers, it is important to be aware that the salt substitute was distributed free of cost during the intervention. Free distribution of the salt substitute was identified as one of the key reasons for the acceptability of the product. To encourage the use of a new product, the group recommended that the government should subsidize it in order to promote its consumption.

1. **Improving the Control of Hypertension in Rural India (CHIRI): Overcoming barriers to diagnosis and effective treatment**

Project dates (January 2014 – October 2016); Publication dates: (May 2018)

*Theoretical Approach: Kirkpatricks’ four level evaluation model*

Kirkpatrick has developed an evaluation guide to help in assessing effectiveness of training programs at four levels: the degree to which participants react favorably, the degree to which participants acquire the intended knowledge and skills, the degree to which participants apply what they have learned and the degree to which targeted outcomes occur. In the CHIRI study, investigators trained Accredited Social Health Activists (ASHAs) to support people with hypertension and teach them how to adopt self-management behaviours for their hypertension. The intervention shifted tasks relating to the measurement of blood pressure and education about risk factors and lifestyle changes from busy primary care physicians to an ASHA, and the management of hypertension to an ASHA and doctor team. ASHAs, were women with at least grade 8 education, chosen through a government approved process involving village leaders and community groups. This category of health worker was already available within India’s National Health Mission, and so was supported by policy.

Process evaluation of the intervention was aimed at: (a) understanding the extent to which the interventions were implemented as planned (fidelity) and (b) understanding the factors that may have impacted the outcomes of the intervention. This case study focuses on the process evaluation of the ASHA’s training program component, undertaken using Kirkpatrick’s four level evaluation model. This was a mixed methods approach incorporating qualitative data collected through interviews, focus group discussions and reports of meetings, and quantitative data collected through fidelity data from meetings.

Training ASHAs for management of hypertension was feasible and led to change in knowledge, skills and motivation. ASHAs delivered the intervention appropriately. ASHAs felt empowered and motivated to perform the tasks they were trained to do. The ASHAs reported that the training materials were easy to understand and useful in educating community members. Interviews with ASHAs indicated that they had developed a better understanding about hypertension and had improved their skills in clinical anthropometric measurement. The community members were appreciative of their new role. Some ASHAs also complained about insufficient remuneration and the lack of supportive supervision and assistance from their line managers within the public health system.

Findings emphasised the need for culturally appropriate training materials for NCDs and their risk factors which can be delivered using interactive and innovative methods. Changes in the health system, such as career opportunities for ASHAs, performance based incentives delivered on time, and innovative training techniques would help improve the morale of this rural community based health workforce. Conducting the mixed-methods process evaluation provided in-depth understanding of the implementation of the program. It was challenging to collect data from three diverse sites and conduct the process evaluation using similar methods. While analysing data, researchers understood the context of each site including the differences in culture and health systems.

1. **Early use of low-dose triple combination of BP lowering drugs in improving BP control in Sri Lanka**

Project dates (February 2014 - December 2017); Publication dates: (August 2018)

*Theoretical Approach: Framework Analysis Method*

In Sri Lanka, a RCT pragmatic study called TRIUMPH was conducted in outpatient departments of 11 urban hospitals investigating effectiveness of early use of low-dose triple combination of BP lowering drugs compared to usual care in improving BP control ^15^. In the trial, randomised patients received either the triple therapy pill from the trial centres or continued with usual care. The RCT is complemented by a formal economic evaluation to assess cost effectiveness and process evaluation to access acceptability of the intervention.

The process evaluation consists of: (a) exploring factors and their interplay that underpin the results of TRIUMPH RCT, (b) exploring patients and providers experience of the intervention, and (c) exploring perceived barriers and facilitators to implementing the intervention in clinical practice. The process evaluation involved face-to-face semi-structured in-depth interviews with a purposive sample of TRIUMPH RCT participants and health care professionals. Trial participants were interviewed at the end of trial as they came off treatment at 6 months follow-up ^16 17^. A multi-disciplinary team, including, clinicians, pharmacists, public health and social scientists were interviewed at the end of follow-up of trial participants.

Patients and providers liked the triple pill because of its ease of use (single pill, once a day dosing) and significant BP control. At the beginning of the trial, providers expressed apprehension about initiating treatment with the triple pill in treatment naïve hypertensive patients. However, over time, they became comfortable about this as no major safety issues were reported and the extent of BP lowering achieved was substantial. Providers expressed a willingness to prescribe the triple pill and patients were willing to use it if it was made available after the trial.

The process evaluation identified many issues that need to be addressed to improve hypertension control in Sri Lanka. Hypertension control programs should include interventions to improve patient education of risk of hypertension and need for long term adherence to drugs to prevent cardiovascular events. Training of prescribers for the management of hypertension particularly using combination therapy coupled with ensuring the availability of combination therapy are likely to improve hypertension control. Interviews with both patients and providers were conducted before the results of the trial was available which helped in exploring views uninfluenced by trial results.

**References**

1. Moore GF, Audrey S, Barker M, et al. Process evaluation of complex interventions: Medical Research Council guidance. *BMJ : British Medical Journal* 2015;350 doi: 10.1136/bmj.h1258

2. Community Matters. Brief introduction to realist evaluation. *Last Accessed on 16/12/2014 Available From:* [*http://wwwcommunitymatterscomau/gpage1html*](http://wwwcommunitymatterscomau/gpage1html) 2004.

3. Medical Research Council United Kingdom. Developing and evaluating complex interventions: new guidance: Available from: [www.mrc.ac.uk/complexinterventionsguidance](http://www.mrc.ac.uk/complexinterventionsguidance). 2009.

4. Webster J, Snowdon W, Moodie M, et al. Cost-effectiveness of reducing salt intake in the Pacific Islands: protocol for a before and after intervention study. *BMC Public Health* 2014;14(1):107. doi: 10.1186/1471-2458-14-107

5. Pawson R, Tilley N. Realistic Evaluation. London, Sage Publications. 1997

6. Fletcher A, Jamal F, Moore G, et al. Realist complex intervention science: Applying realist principles across all phases of the Medical Research Council framework for developing and evaluating complex interventions. *Evaluation (London, England : 1995)* 2016;22(3):286-303. doi: 10.1177/1356389016652743

7. Better Evaluation. Realist Evaluation. *Last Accessed 16/12/2014 Available From:* [*http://betterevaluationorg/approach/realist_evaluation*](http://betterevaluationorg/approach/realist_evaluation) 2014

8. Thorogood M, Goudge J, Bertram M, et al. The Nkateko health service trial to improve hypertension management in rural South Africa: study protocol for a randomised controlled trial. *Trials* 2014;15:435. doi: 10.1186/1745-6215-15-435 [published Online First: 2014/11/09]

9. Maar MA, Yeates K, Perkins N, et al. A Framework for the Study of Complex mHealth Interventions in Diverse Cultural Settings. *JMIR mHealth and uHealth* 2017;5(4):e47. doi: 10.2196/mhealth.7044

10. Maar MA, Yeates K, Toth Z, et al. Unpacking the Black Box: A Formative Research Approach to the Development of Theory-Driven, Evidence-Based, and Culturally Safe Text Messages in Mobile Health Interventions. *JMIR mHealth and uHealth* 2016;4(1):e10. doi: 10.2196/mhealth.4994

11. Maar M, Yeates K, Barron M, et al. I-RREACH: an engagement and assessment tool for improving implementation readiness of researchers, organizations and communities in complex interventions. *Implementation Science : IS* 2015;10:64. doi: 10.1186/s13012-015-0257-6

12. Kane SS, Gerretsen B, Scherpbier R, et al. A realist synthesis of randomised control trials involving use of community health workers for delivering child health interventions in low and middle income countries. *BMC Health Serv Res* 2010;10:286. doi: 10.1186/1472-6963-10-286 [published Online First: 2010/10/15]

13. Vedanthan R, Kamano JH, Naanyu V, et al. Optimizing linkage and retention to hypertension care in rural Kenya (LARK hypertension study): study protocol for a randomized controlled trial. *Trials* 2014;15:143-43. doi: 10.1186/1745-6215-15-143

14. Bernabe-Ortiz A, Diez-Canseco F, Gilman RH, et al. Launching a salt substitute to reduce blood pressure at the population level: a cluster randomized stepped wedge trial in Peru. *Trials* 2014;15:93-93. doi: 10.1186/1745-6215-15-93

15. Salam A, Webster R, Singh K, et al. TRIple pill vs Usual care Management for Patients with mild-to-moderate Hypertension (TRIUMPH): Study protocol. *American Heart Journal* 2014;167(2):127-32. doi: <https://doi.org/10.1016/j.ahj.2013.10.020>

16. Patton MQ. Qualitative evaluation and research methods: SAGE Publications, inc; 1990.

17. Bazeley P. Qualitative data analysis: Practical strategies: Sage; 2013.
